# Down Syndrome Reduces the Sedative Effect of Midazolam in Pediatric Cardiovascular Surgical Patients

**DOI:** 10.1038/s41598-020-58283-1

**Published:** 2020-02-10

**Authors:** Yujiro Matsuishi, Hideaki Sakuramoto, Haruhiko Hoshino, Nobutake Shimojo, Yuki Enomoto, Bryan J. Mathis, Yuji Hiramatsu, Yoshiaki Inoue

**Affiliations:** 1 Department of Emergency and Critical Care Medicine, Faculty of Medicine, University of Tsukuba, Tsukuba, Ibaraki Japan; 2 Adult Health Nursing, College of Nursing, Ibaraki Christian University, Hitachi, Ibaraki Japan; 3 Department of Pediatrics, University of Tsukuba Hospital, Tsukuba, Ibaraki Japan; 4 Medical English Communication Center, Faculty of Medicine, University of Tsukuba, Tsukuba, Ibaraki Japan; 5 Department of Cardiovascular Surgery, University of Tsukuba, Tsukuba, Ibaraki Japan

**Keywords:** Paediatrics, Paediatric research

## Abstract

Down syndrome (DS) is frequently comorbid with congenital heart disease and has recently been shown to reduce the sedative effect of benzodiazepine (BDZ)-class anesthesia but this effect in a clinical setting has not been studied. Therefore, this study compared midazolam sedation after heart surgery in DS and normal children. We retrospectively reviewed patient records in our pediatric intensive care unit (PICU) of pediatric cardiovascular operations between March 2015 and March 2018. We selected five days of continuous post-operative data just after termination of muscle relaxants. Midazolam sedation was estimated by Bayesian inference for generalized linear mixed models. We enrolled 104 patients (average age 26 weeks) of which 16 (15%) had DS. DS patients had a high probability of receiving a higher midazolam dosage and dexmedetomidine dosage over the study period (probability = 0.99, probability = 0.97) while depth of sedation was not different in DS patients (probability = 0.35). Multi regression modeling included severity scores and demographic data showed DS decreases midazolam sedation compared with controls (posterior OR = 1.32, 95% CrI = 1.01–1.75). In conclusion, midazolam dosages should be carefully adjusted as DS significantly decreases midazolam sedative effect in pediatric heart surgery patients.

## Introduction

Down syndrome (DS), or trisomy 21, is the most common chromosome disorder^1^ with a birth prevalence estimated at 1.5 per 1,000^2^ and approximately 5,000 children are born with DS in the United States each year^3^. Similarly, a previous regional survey of Japan reported a prevalence of 1.5 per 1,000 live births^4^. Patients with DS frequently have associated developmental disorders and about 40% of children with DS are born with congenital heart disease (CHD)^5^, leading to higher mortality rates. Although many of these malformations can be surgically corrected, DS patients face additional risks such as airway obstruction while under sedation^6^. Recently, pharmacological interactions of dexmedetomidine (DEX), a highly selectiveα2-adrenergic agonist, with DS patients were shown to result in more side effects^7^. Additionally, benzodiazepine (BDZ), which increases GABA_A_ receptor-mediated chloride ion influx, is thought to engender pharmaco-resistance in DS patients due to altered GABAergic transmission in area CA1^8^ as seen in murine models. The manifestation of this effect has been clinically seen in case reports showing resistance to BDZ-class anesthesia midazolam (MDZ) in DS patients, such as dental surgery in a 35-year-old DS patient that required 3.5 mg MDZ before local anesthesia^9^. However, statistical confirmation of this effect in DS patients remains elusive^10^. Therefore, this study was conducted under the hypothesis that MDZ sedative effect is lessened in DS patients and aimed to use a sufficient sample size to discover the impact of MDZ resistance in pediatric surgery.

## Methods

### Study design and participants

We retrospectively reviewed records of 131 consecutive patients admitted to the University of Tsukuba Affiliated Hospital pediatric intensive care unit (PICU) who underwent cardiovascular operations between March 2015 and March 2018. Patients were excluded if they had other trisomy, stroke, epilepsy and/or PICU stays of less than 5 days after the end of muscle relaxant usage. We recorded patient information, including age, sex, surgical procedure, and daily severity data (including severity of organ dysfunction and sedative/muscle relaxant dosages) during PICU stays for five days after the end of muscle relaxant usage. In our practice, we use muscle relaxants and sedation in cases of severe cardiac failure and pulmonary arterial hypertension. Additional instances would be whenever careful control is needed for a stressed right ventricle (such as for the Fontan procedure) and/or to prevent fighting the ventilator and reduce oxygen consumption. We also use muscle relaxants and sedation for high airway resistance patients, but all uses of relaxants and sedation are carefully monitored and weaning is judged on both a daily and case-by-case basis. The Institutional Review Board of the University of Tsukuba approved the study (Approval #H29-134).

### Evaluation tools

The severity of cardiovascular procedures was evaluated by Risk Adjustment in Congenital Heart Surgery (RACHS-1)^11^ which classifies surgical procedures into six categories based on mortality risk. RACHS-1 was previously validated by large multi-institutional data sets^12–14^. Sedation was assessed by using the State Behavioral Scale (SBS)^15^, which scores sedation status over a range of −3 (unresponsive) to +2 (agitation), and is widely used in the pediatric critical care field as a sedation indicator^16^. Daily severity of organ dysfunction was evaluated by PEdiatric Logistic Organ Dysfunction-2 score (PELOD-2)^17^. PELOD-2 consists of ten variables corresponding to five organ dysfunctions and daily assessment allows for prediction of outcome in critically ill children^18^.

### Statistical analysis

#### Model structure

The outcome of interest was sedation status measured by SBS and the dependent factor was MDZ dosage. However, muscle relaxants are ordinarily used after cardiac operations to avoid negative hemodynamic effects and this may mask both sedation status and pharmaco-resistance to sedatives. However, excluding muscle relaxant usage days would cause lead time bias and possible overestimation of the outcome^19,20^. Thus, we used muscle relaxant days as a covariate for the multi-regression modeling to mediate the bias in addition to a five-day post-usage period in our analysis. For adjustment of our model, additional covariates were chosen *a priori*: sex, age, RACHS-1, dose of dexmedetomidine, vecuronium dosage status (received/not received) and PELOD-2 (without the central nervous system component). To adjust patient demographic characteristics, we chose sex and age as covariates. RACHS-1 was used to adjust operation severity and PELOD-2 (without the central nervous system component) was used to adjust post-surgical daily severity. As our institute mainly uses dexmedetomidine as MDZ alternatives for sedation, we chose dosages of this as covariate factors.

#### Interaction methodology

Our modeling included considerations about interaction. As regression modeling assumes independence for each factor, we suspected that Down syndrome and MDZ dosage were not independent of each other and the magnitude (quantitative interaction) of MDZ effect would change based on DS status. Thus, we used a two-step system in which we calculated main effect (model 1) then proceeded to interaction modeling (model 2). We also applied an interaction methodology for DEX (model 3) as a control for midazolam pharmaco-resistance to sedation. In interaction models (model 2, 3) the odds ratio of the main effect (Down syndrome and midazolam; Down syndrome and dexmedetomidine) was not significant, possibly due to the ability to capture only a segment of the main effect.

#### Statistical estimation

Surveying for pharmaco-resistance assumes the inclusion of many outliers. A previous study already reported using robust methods in multivariate methods while Bayesian methods^21,22^ are also applicable for data that includes many outliers. To deal with population outliers in pharmaco-resistance studies, Bayesian modeling^23,24^ and Bayesian inference for generalized linear mixed models (GLMM) via Markov chain Monte Carlo (MCMC) has been reported^25,26^. Therefore, we applied Hierarchical Bayesian Modeling, or Bayesian inference for GLMM using No-U-turn sampler (NUTS), which is an extension of the Hamiltonian Monte Carlo (HMC) algorithm of the Markov chain Monte Carlo method^27^. We used uninformative prior distribution as our prior distribution and all iterations were set to 2,000, burn-in samples were set to 1,000 and the number of chains was set to 4. To check the modeling assumption, we used the value of Rhat, Monte Carlo Standard Error (MCSE)/standard deviation (sd), and effective sample size (Neff)/numbers (N). An MCSE/sd less than 10%, a Neff/N more than 10%, and a Rhat for all parameters less than 1.1^28^ indicated a good estimation for the model. We report the 95% percentile interval as a 95% credible interval (Crl). We also report the probability for supporting the hypothesis as greater or less than another group in univariate analysis.

#### Ethics approval and consent to participate

We used an opt-out methodology coupled with informed consent for this study that was approved by the Institutional Review Board of the University of Tsukuba (Approval # H29-134). Information about the study (study goals, methods, and the right to opt out at any point in the study) was available online and in printed form at the hospital. All procedures were approved under regulations of the University of Tsukuba that equal or exceed the standards set by the Declaration of Helsinki.

## Results

### Patient characteristics

**Figure 1:**
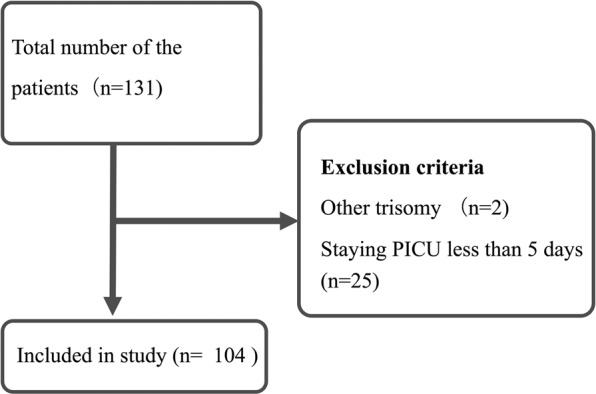
Participant flow chart. Participant flow chart. This figure shows participant flow chart including exclusion criteria, and final enrollment patients for the investigation.

**Figure 2:**
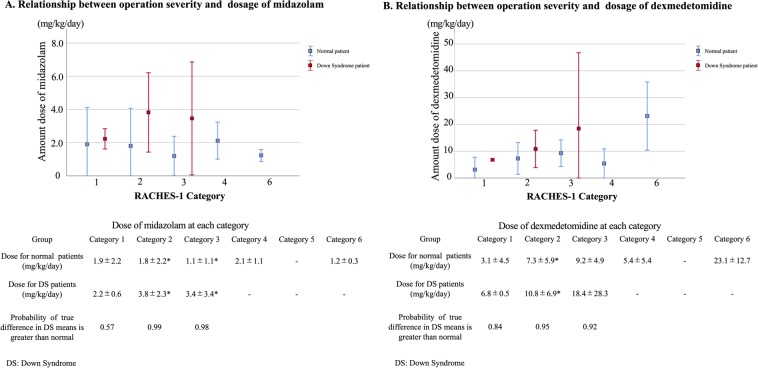
Relationship between operation severity and sedative dosage. The figure shows the relationship between operation risk score measured by RACHES-1 and sedative dosage. Category 1 operations tended to not differ in total amounts of sedative, but Category 2 and Category 3 had a high probability that DS patients would need a higher total amount of sedative (the probability of the DS group dosage of midazolam was greater than normal: Category 1 = 0.57, Category 2 = 0.99, Category 3 = 0.98; the probability of the DS group dosage of dexmedetoimidine was greater than normal: Category 1 = 0.84, Category 2 = 0.95, Category 3 = 0.92).

Two patients with another form of trisomy were excluded from this study. There were no instances of stroke and epilepsy but 25 patients were excluded for a PICU stay of less than 5 days. We analyzed a total 520 data points from 104 patients for this study (Fig. 1). Table 1 presents the demographic characteristics of enrolled patients. Similar numbers of male and female DS patients were enrolled in this study (50% vs 48% in controls; probability of DS female prevalence was greater than normal = 0.5). Average age of the total population was 26 (± 40) weeks and DS patients had a high probability to be younger than normal patients (12 ± 22 weeks vs. 28 ± 42 weeks, respectively; probability of the DS group mean was greater than normal = 0.93) while muscle relaxant days had a high probability to be longer for DS patients (3 days in DS vs 0 days in controls; probability of the DS group’s days were greater than normal = 0.97). RACHS-1 scores were almost identical in the DS patients compared with normal patients (2 in DS vs 2 in controls; probability of the DS group’s severity was greater than normal = 0.13) and PELOD-2 found that DS patients had a higher probability to be younger than normal patients (5.6 ± 1.9 in DS vs. 4.1 ± 1.8 in controls; probability of DS group severity was greater than normal = 0.99). Depth of sedation was one area where DS patients did not have a high probability compared with normal patients over the study period (−1 in DS vs −1 in controls; probability of DS group mean was greater than normal = 0.35) but DS patients had a high probability of receiving significantly higher doses of midazolam, dexmedetomidine and Fentanyl (midazolam: 3.5 mg/kg/day in DS vs. 1.6 mg/kg/day in control, probability of DS group mean was greater than normal = 0.99; dexmedetomidine:11.7 μg/kg/day in DS vs. 7.6 μg/kg/day in control, probability of DS group mean was greater than normal = 0.99; Fentanyl: 0.9 mg/kg/day in DS vs. 0.1 mg/kg/day in control, probability of DS group mean was greater than normal = 0.99). Figure 2 presents the relationship between operation risk score as measured by RACHES-1 and sedative dosage amounts. Category 1 operations tended to not differ in total amounts of sedative, but Category 2 and Category 3 had a high probability that DS patients would need a higher total amount of sedative (the probability of the DS group dosage of midazolam was greater than normal: Category 1 = 0.57, Category 2 = 0.99, Category 3 = 0.98; the probability of the DS group dosage of dexmedetoimidine was greater than normal: Category 1 = 0.84, Category 2 = 0.95,Category 3 = 0.92).

**Table 1:** Baseline characteristics of study patients.

variable	Total population N = 104	Down Syndrome N = 16	Normal N = 88	Hypothesis for the estimation	Probability^a^
Age, weeks, ±SD	26 ± 40	12 ± 22	28 ± 42	True difference in DS mean is less than normal	0.93
Female, n, (%)	51 (49)	8 (50)	43 (48)	True difference in DS prevalence is greater than normal	0.50
RACHS-1^b^, score, ±SD	2 (2, 2)	2 (2, 2)	2 (1, 2)	True difference in DS severity is greater than normal	0.13
Category 1	10	2 (20%)	8 (80%)		
Category 2	66	11 (16%)	55 (84%)		
Category 3	22	3 (14%)	19 (86%)		
Category 4	4	—	4 (100%)		
Category 5	—	—	—		
Category 6	2	—	2 (100%)		
PELOD 2^c^, score, ±SD	4.3 ± 1.8	5.6 ± 1.9	4.1 ± 1.8	True difference in DS severity is greater than normal	0.99
SBS^d^, score, (IQR)	−1 (−2, −1)	−1 (−2, −1)	−1 (−2, −1)	True difference in DS depth of sedation is less than normal	0.35
Midazolam, mg/kg/day ± SD	1.9 ± 2.1	3.5 ± 2.3	1.6 ± 2.0	True difference in DS means is greater than normal	0.99
Dexmedetomidine, μg/kg/day, ±SD	8.2 ± 7.6	11.7 ± 12.3	7.6 ± 6.2	True difference in DS means is greater than normal	0.97
Fentanyl, mg/kg/day, ±SD	0.2 ± 1.0	0.9 ± 2.4	0.1 ± 0.4	True difference in DS means is greater than normal	0.99
Muscle relaxant days, days, (IQR)	0 (0, 2)	3 (0, 5)	0 (0, 3)	True difference in DS means is greater than normal	0.97

^a^Using Bayesian t-test or Bayesian AB test.

^b^RACHS-1 = Risk-Adjusted Congenital Heart Surgery-1.

^c^PELOD2 = Pediatric Logistic Organ Dysfunction 2, without the central nervous system component.

^d^SBS = State Behavioral Scale.

IQR = interquartile range; SD = standard deviation.

### Multi regression modeling

The MCSE/sd was less than 10%, the Neff/N more than 10%, and Rhat for all parameters was less than 1.1. Therefore, our model was a good fit for estimation and did not violate any assumptions.

**Figure 3:**
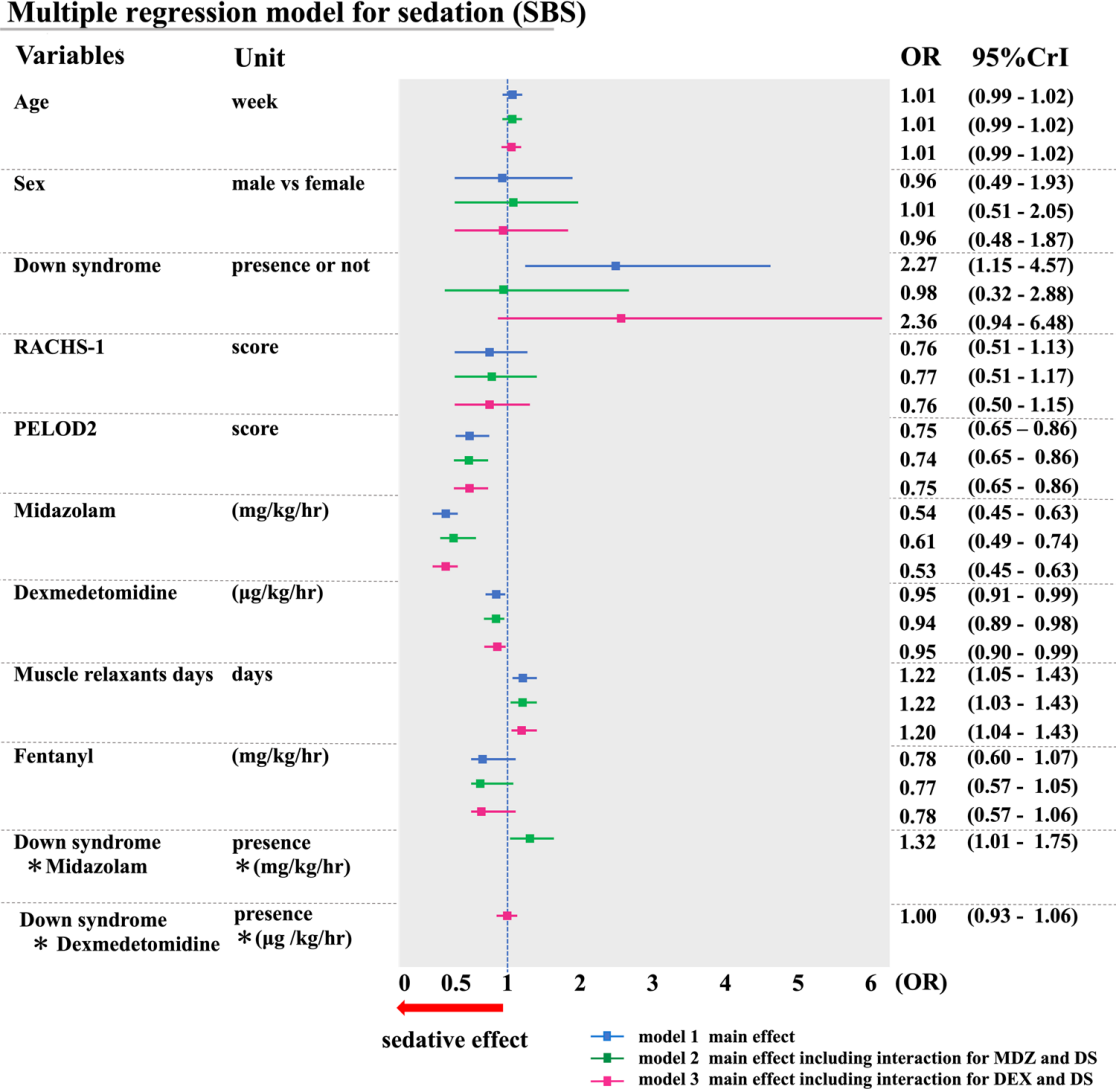
Figure of multiple regression model for sedation. The figure shows the main effect for model 1 in blue, the main effect (including interaction for midazolam [MDZ] and down syndrome [DS] interaction) for model 2 in green and the main effect in model 2 for dexmedetomidine (DEX) in red. An posterior odds ratio (OR) less than 1.0 indicates that a factor has a sedative effect. All the models estimate sedative effect by using 520 continuous data points from 104 patients.

**Figure 4:**
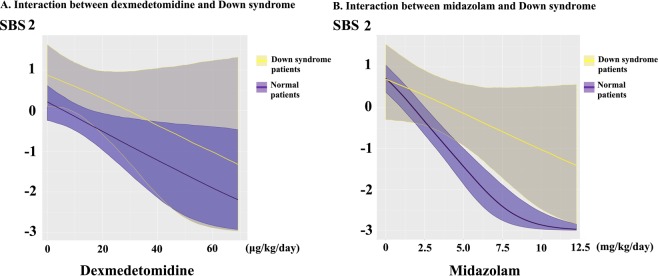
Interaction between sedatives and Down syndrome. The figure shows the interaction of sedative effect of dexmedetomidine (DEX) and midazolam (MDZ) for normal and down syndrome (DS) patients as estimated by Bayesian inference for GLMM using No-U-turn sampler (NUTS). Shaded area indicates 95% credible intervals. (**A**) Describes associations between sedation status as estimated by SBS score and dose of DEX in both DS and normal patients. (**B**) Describes association between sedation status as estimated by SBS score and dose of MDZ in both DS and normal patients.

Figure 3 shows results from multi regression modeling. In our main effect model (model 1), Down syndrome [posterior odds ratio (OR) = 2.27, 95% credible interval (CrI) = 1.15–4.57] and muscle relaxants days (posterior OR = 1.22, 95% CrI = 1.05–1.43) are positively associated with an arousal effect. PELOD-2 score (posterior OR = 0.75, 95% CrI = 0.65–0.86), midazolam (posterior OR = 0.54, 95% CrI = 0.45–0.63) and dexmedetomidine (posterior OR = 0.95, 95% CrI = 0.91–0.99) were positively associated with a sedative effect. Our interaction modeling for MDZ (model 2) found that interaction between MDZ and DS was positive (posterior OR = 1.32, 95% CrI = 1.01–1.75), indicating that the sedative effect of midazolam is decreased in DS patients compared to control patients (posterior OR = 1.32, 95% CrI = 1.01–1.75) (model 2) while interaction modeling for DEX was not (posterior OR = 1.00, 95% CrI = 0.93–1.06) (model 3) (Fig. 4).

## Discussion

This retrospective study aimed to evaluate factors that could contribute to differences in MDZ sedative effect between DS and control patients. A total of 104 pediatric patients in the PICU after cardiac surgery were enrolled and evaluated using validated tools over 5 consecutive days. We found that, overall, the amount of MDZ administered was increased in DS versus controls after ending muscle relaxants and observed the reduced sedative effect of MDZ for DS patients while DEX was not different as estimated by Bayesian inference modeling. These results are in line with previous research which showed higher requirements for MDZ in neonatal cardiac surgery in DS patients^10^.

Researching sedative effects in a minority population (such as in pediatric DS patients) is complicated from bias imparted by heterological prevalence and complications. The demographic data and operation risk of this study is also heterological between normal patients and DS patients. To adjust these biases, we used multivariate analysis with respect to these factors but we also “double checked” our results by using the demographic data propensity score for DS patients as a covariate in Bayesian inference modeling. The result (Table 2) also shows a reduced sedative effect of MDZ while DEX was not different.

**Table 2:** Multiple Regression model for sedation (SBS) using propensity score.

	Multivariate model 1 posterior OR (95% CrI)^a^	Multivariate model 2 posterior OR (95% CrI)^a^
Propensity score^b^	5.75 (0.39–90.9)	3.18(0.20–51.4)
Down syndrome (presence or not)	0.83 (0.17–3.8)	2.97(0.77–11.1)
Midazolam (mg/kg/hr)	0.49(0.41–0.58)	0.53(0.45–0.62
Dexmedetomidine (μg/kg/hr)	0.92(0.88–0.96)	0.93(0.89–0.98)
Muscle relaxants days (days)	1.16(0.99–1.36)	1.16(0.99–1.33)
Fentanyl (mg/kg/hr)	0.78(0.58–1.05)	0.80(0.60–1.07)
PELOD2^c^	0.74(0.65–0.84)	0.74(0.65–0.85)
Down syndrome presence *Midazolam *(mg/kg/hr)	1.63 (1.11–2.36)	—
Down syndrome presence *Dexmedetomidine *(μg/kg/hr)	—	1.0(0.93–1.10)

^a^Model estimated by Bayesian inference for GLMM using No-U-turn sampler (NUTS) The MCSE/sd was less than 10%, the Neff/N more than 10%, and Rhat for all parameters was less than 1.1.

^b^Sex, age, RACHS-1 was used for estimate propensity score for Down syndrome.

^c^PELOD2 = Pediatric Logistic Organ Dysfunction 2, without the central nervous system component.

Pediatric heart surgery is a complex process complicated by DS. Although some studies showed no differences in mortality between DS and normal pediatric patients, it is in the recovery stage that DS complications arise^29,30^. Recovery from heart surgery is difficult even in adult patients and for pediatric cases complicated by DS, recovery troubles are compounded by various developmental deficits^7^. A retrospective study by Nasser and colleagues found that almost 12% of DS patients recovering from heart surgery needed prolonged mechanical ventilation and almost half of these patients required medication for resultant hypertension^31^. Hematological abnormalities can also be present, complicating wound healing^32,33^. Adding to this complex issue is the amount of sedative needed to prevent unnecessary suffering and anxiety during the healing process. In DS patients, there is clinical evidence that trisomy disorders increase the need for MDZ and similar drugs due to alterations in the GABAergic transmission system^10,34^. On the translational side, many fundamental studies showed altered GABAergic transmission in murine models that mimic the brain morphology of DS^34^ and indicated that GABA_A_ receptor-mediated synaptic transmission occurs in the hippocampus^35^.The propensity of DS patients to more frequently suffer from epilepsy (mediated in the hippocampus), along with dysregulated GABA excitatory-inhibitory balance is hypothesized to be one of the main reasons for the reduced effect of BDZ^36^. With this hypothesis in mind, we sought to establish a more solid link between DS and BDZ-class anesthesia midazolam requirements by a retrospective, single-center analysis. Although we did not do genetic screens, we did have validated sedation and risk scales (SBS and PELOD-2) to base our measurements on. We compensated for fluctuations in recovery inherent to cardiovascular surgery by using Bayesian inference for GLMM. We found that, in general, DS patients required more BDZ over longer periods than normal patients and that daily cumulative doses of dexmedetomidine were increased to compensate for the lessened effect of MDZ. Although we did not observe any prevalence of sedation-related syndromes such as withdrawal and delirium over our study period, a longer duration, multi-centered study saw a prevalence of withdrawal syndrome of more than 60% after mechanical ventilation and sedative administration of more than 5 days^37^. As DS patients have more complex recoveries than normal, it is entirely possible that any recovery lengthened by DS complications will require more sedatives that, in turn, will increase withdrawal symptoms after prolonged usage. Furthermore, a recent study showed that MDZ usage is a risk factor for developing pediatric delirium^38^. Taken together, these results pair well with our results and indicate that a new approach to sedation in DS patients is required. Unpredictable, variable and serious complications in respiration, clotting, drug metabolism, and slower healing after surgery require longer recoveries and more sedation to compensate for this. Additionally, as DS patients may suffer from withdrawal symptoms after extended use of sedatives such as BDZ, this could introduce additional suffering to patients already weakened from invasive surgery.

To conclude, we conducted a retrospective study based on validated evaluative tools that indicated a need for higher doses of MDZ with higher doses of compensating sedatives for the 5-day period immediately after muscle relaxant usage following pediatric heart surgery. Our results indicate a need for careful monitoring of sedative effect as DS patients may not respond to MDZ in a satisfactory manner. Non-BDZ sedatives (such as Z-drugs) that reduce pain and avoid potential troubles such as withdrawal and delirium need to be evaluated with respect to trisomy disorders and pediatricians should work carefully with pain management specialists to ensure that patient needs are effectively met until alternate approaches are validated.

## Limitations

There is some limitation that our retrospective design did not validate patient genetics but relied solely on recorded data. This could affect accuracy and there is variability in the evaluative tools we used due to the subjective nature of scales like the SBS. However, in spite of being a single-center study, we believe our patient pool was of sufficient size and power to maintain significance for our main result and our results were similar to other studies in the field. Moreover, as we used 520 data points collected over 5 days from 104 patients and we adhered to a balanced design that ensured all participants had the same number of data points at each level, we are confident in the validity of our results. Our statistical method was chosen based on a two-step process to take into account the magnitude of interaction with MDZ but the original assumption of the independence of all factors may be correct. In this case, however, the results from our first modeling step would still be valid. In spite of the limitations inherent in a single-center, retrospective study, this report still shows a significant link between DS and altered BDZ effect that could serve to bring insight into clinical practice and act as a basis for controlled, clinical studies.

## Conclusion

We revealed MDZ’s quantitative interaction for sedative effect with and without DS in clinical care settings and MDZ dosages should be carefully adjusted in pediatric heart surgery patients.

## Data Availability

The datasets used and/or analyzed during the present study are available from the corresponding author upon reasonable request.
